# HIV seroconversion and associated factors among seronegative pregnant women attending ANC in Ethiopia: an institution-based cross-sectional study

**DOI:** 10.3389/frph.2024.1246734

**Published:** 2024-04-10

**Authors:** Dawit Sisay Dessale, Mulugeta Betre Gebremariam, Asrat Arja Wolde

**Affiliations:** ^1^ School of Public Health, College of Health Sciences, Addis Ababa University, Addis Ababa, Ethiopia; ^2^ National Data Management and Analytics Center, Ethiopian Public Health Institute, Addis Ababa, Ethiopia

**Keywords:** (ANC) antenatal care, (HIV) human immunodeficiency virus, (ART) antiretroviral therapy, (PMTCT) prevention of mother-to-child transmission, (STI) sexually transmitted infections, (VCT) voluntary counseling and testing, (UNAIDS) United Nations Program on HIV/AIDS

## Abstract

**Background:**

In countries with limited resources, including Ethiopia, HIV is diagnosed using a rapid serological test, which does not detect the infection during the window period. Pregnant women who test negative for HIV on the first test may seroconvert throughout pregnancy. Women who are seroconverted during pregnancy may not have received interventions, as they are considered HIV-negative unless they are retested for HIV at the end of their pregnancy. Due to limited data on HIV seroconversion, this study aimed to measure the extent of HIV seroconversion and to identify associated factors among seronegative pregnant women attending ANC in Ethiopia.

**Methods:**

Institution-based cross-sectional study was conducted among HIV-negative pregnant women attending the ANC in Ethiopia between June and July 2020. Socio-demographic, clinical, and behavioral data were collected through face-to-face questionnaires and participants' records review. HIV retesting was performed to determine the current HIV status of pregnant women. The data collected were entered into Epi data version 4.4.1 and were exported and analyzed by SPSS version 25. A *p*-value < 0.25 in the bivariate analysis was entered into multivariable logistic regression analysis and a *p*-value of < 0.05 was considered statistically significant.

**Result:**

Of the 494 pregnant women who tested negative for HIV on their first ANC test, six (1.2%) tested positive on repeat testing. Upon multivariable logistic regression, pregnant women who have had a reported history of sexually transmitted infections [AOR = 7.98; 95% CI (1.21, 52.82)], participants' partners reported travel history for work frequently [AOR = 6.00; 95% CI (1.09, 32.99)], and sexually abused pregnant women [AOR = 7.82; 95% CI (1.194, 51.24)] were significantly associated with HIV seroconversion.

**Conclusion:**

The seroconversion rate in this study indicates that pregnant women who are HIV-negative in early pregnancy are at an ongoing risk of seroconversion throughout their pregnancy. Thus, this study highlights the benefit of a repeat HIV testing strategy in late pregnancy, particularly when the risk of seroconversion or new infection cannot be convincingly excluded. Therefore, repeated testing of HIV-negative pregnant women in late pregnancy provides an opportunity to detect seroconverted pregnant women to enable the timely use of ART to prevent mother-to-child transmission of HIV infection.

## Introduction

Vertical transmission of human immunodeficiency virus (HIV) remains a public health problem worldwide, particularly in less developed countries (1). Without intervention, the risk of mother-to-child transmission (MTCT) of HIV can range from 15 to 45%. However, the risk can be reduced to less than 1% through appropriate interventions (1, 2). According to the Ethiopian Demographic and Health Survey 2016, the national HIV prevalence is 0.9% (3). More than 90% of HIV infections in children occur during pregnancy, childbirth, and breastfeeding (4, 5). Several individual studies suggest that the incidence of HIV infection during pregnancy is high. The sexual behavior of the woman or her partner and physiological changes during pregnancy are possible mechanisms that explain the increased susceptibility of women to HIV infection during pregnancy (6). In addition, structural changes in the mucosa of the genital tract or immunological effects caused by high levels of estrogen and progesterone during pregnancy may increase the risk of HIV infection in women (7, 8). HIV also causes additional physical, immunological, and psychological stress during pregnancy and increases maternal and neonatal morbidity and mortality (9).

According to a 2018 systematic review and meta-analysis, the cumulative HIV prevalence among pregnant women in Ethiopia was 5.74% (10). Another meta-analysis conducted in Ethiopia found that 9.9% of children were infected with HIV from their mothers due to a lack of interventions, suggesting that HIV infection remains a public health problem in the country (11). A number of studies have demonstrated that pregnant women who initially test negative for HIV may subsequently test positive. Being sexually active during undue behavioral drives, physiological and biological changes, pregnant women remain at risk of HIV seroconversion and transmission of the virus to their newborns (12, 13). These studies also found that history of sexually transmitted infections, polygamy, alcohol consumption, women's age, history of partners with sexually transmitted infections, women with multiple sexual partners, HIV-positive partner, level of education, shortest relationship, occupational status and knowledge about HIV as a risk factor for HIV seroconversion (11–18).

Several approaches have been developed in the past three decades to prevent mother-to-child transmission of HIV (PMTCT), including early detection of HIV-infected pregnant women and initiation of antiretroviral (ARV) therapy (5). The fact that the number of new HIV infections among children worldwide has fallen from 310,000 in 2010 to 130,000 in 2022 shows how valuable these services are (19). While approximately 2.5 million new HIV infections have been averted since the introduction of PMTCT services, approximately 180,000 new infections were reported in 2017, the majority (90%) of which occurred in sub-Saharan Africa. This indicates that children are being left behind, which is a huge injustice to our children (20–22). Integrating voluntary HIV counseling and testing into antenatal care (ANC) and initiating antiretroviral therapy in pregnant women chronically infected with HIV has proven to be a very effective strategy. However, women who are infected with HIV or who seroconvert during pregnancy may go undetected and untreated unless they are retested in late pregnancy. Although programs focus on preventing mother-to-child transmission of HIV in chronically infected women, pregnant women who have recently become infected with HIV may still contribute to HIV transmission to the fetus (23–25).

In resource-limited countries, including Ethiopia, HIV is diagnosed using a rapid serological test that does not detect the infection during the window period. Pregnant women who have recently had an infection or who have been seroconverted do not have an extra window of opportunity unless they are retested. Recently, several countries, including Ethiopia, have recommended repeat HIV testing in late pregnancy as a standard of care as it increases ARV provision to newly infected or seroconverted pregnant women to improve the women's and children's health (24, 26–29). Even while the Ethiopian government recommends repeat HIV testing at the end of pregnancy (30), this recommendation is rarely followed through, and the emphasis is usually on HIV-positive pregnant women. However, primary prevention for HIV-negative women in early pregnancy is often overlooked. Pregnant women who are HIV seronegative early in pregnancy remain at risk of seroconversion throughout pregnancy.

Due to a lack of attention, women who seroconvert during pregnancy may not have received interventions, as they are assumed to be HIV-negative. These particular categories of pregnant women may not even receive counseling on the best method of infant feeding, thereby increasing vertical transmission of HIV (12). Diagnosis and treatment of seroconverted pregnant women are also critical to achieving the UNAIDS vision of eliminating vertical transmission of HIV by 2030 (31).

In the study area, pregnant women are tested for HIV only once during their first visit to the ANC. In contrast, HIV-negative pregnant women do not have tested again at the end of their pregnancy. Due to limited data on HIV seroconversion, this study aimed to measure the extent of HIV seroconversion and to identify associated factors in HIV-negative pregnant women attending ANC visits using a repeat HIV testing strategy at the end of pregnancy.

## Methods and materials

### Study setting

The study was conducted in Kobo Town and Raya Kobo Woreda. The town is 570 kilometers from Addis Ababa and 410 kilometers from Bahirdar. There is one government hospital, one private hospital, one health center, and five health posts in the city. There are also eight health centers and forty-five health posts in Raya Kobo Woreda. In the calendar year 2020, 4,447 pregnant women received early antenatal care, of which 4,245 were tested for HIV, 65 were HIV positive, and were immediately started on antiretroviral therapy (ART).

### Study design and period

An institution-based cross-sectional study was conducted from June 01, 2020, to July 30, 2020, among booked seronegative pregnant women attendees in governmental health facilities.

### Population

#### Source population

All booked HIV seronegative pregnant women found in Kobo Town and Raya Kobo Woreda governmental health facilities.

#### Study population

All booked HIV seronegative pregnant women in the selected governmental health care facilities during the data collection time.

### Inclusion and exclusion criteria

All booked HIV seronegative pregnant women attendees in governmental health care facilities irrespective of their gestational age at booking and have had at least three months between their initial HIV negative test results. However, pregnant women who had severe medical or obstetric complications and mental health problems were excluded from the study.

### Sample size determination

The sample size for the first specific objective was calculated using a single population proportion formula, assuming the previous study's HIV seroconversion rate in Nigeria was 3.9% (32); a margin of error of 2% was assumed, and according to the 95% confidence interval, the calculated sample size was 360, and considering the non-response rate of 10%, the final sample size was 400. For the second specific objective, post-primary education has an adjusted odds ratio of 2.4, and the proportion of cases among the uneducated was 7.7% and adding a non-response rate of 10% the sample size was 509. The calculated sample size for the first specific objective using a single population proportion formula was 400, which is smaller than the calculated sample size for the second specific objective, which is 509. Thus, a total of 509 pregnant women were recruited as study units from among pregnant women who attended antenatal follow-up care at health facilities in Kobo Town and Raya Kobo Woreda during the study period.

### Sampling procedure

**Figure 1:**
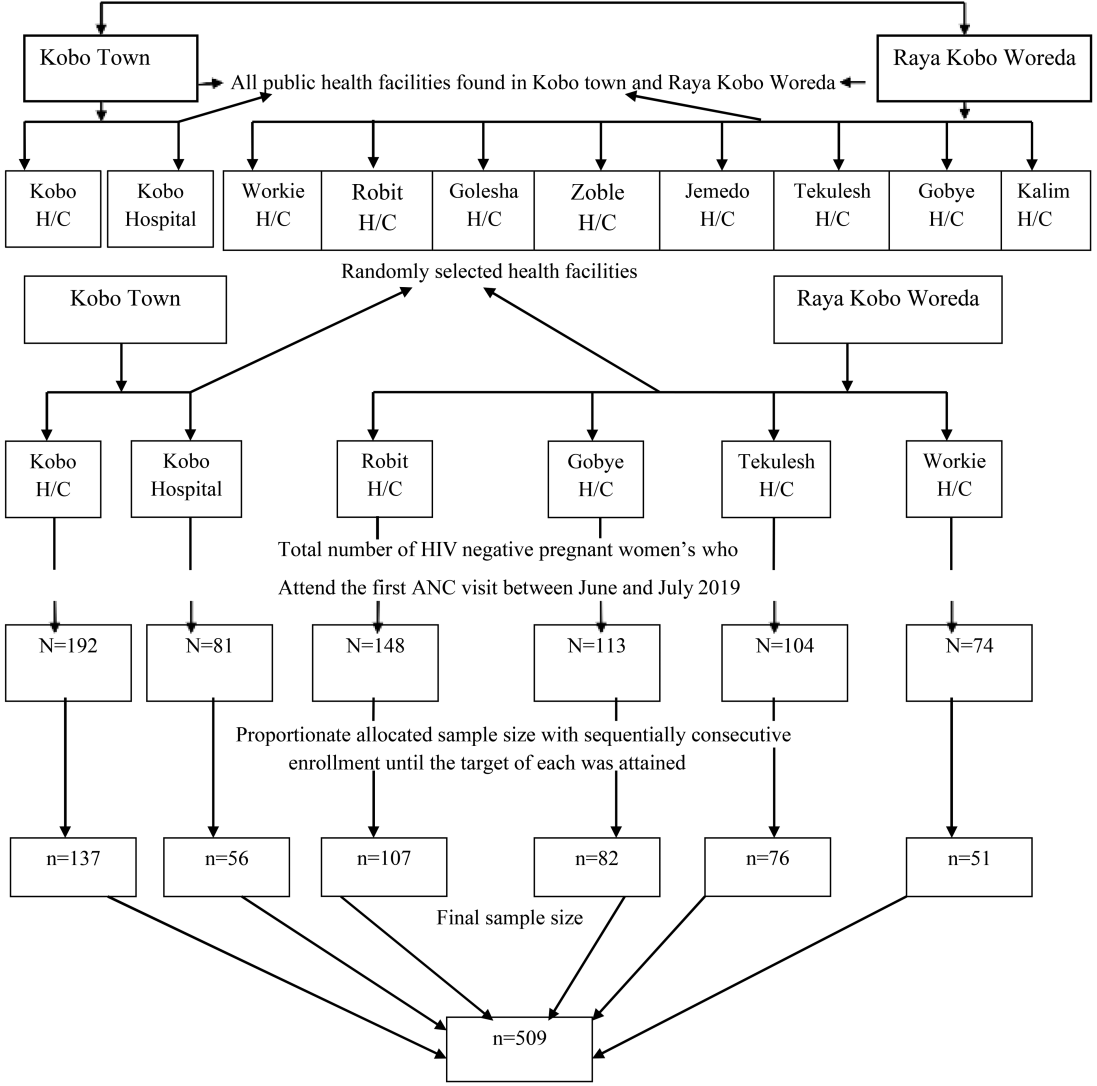
Schematic representation of the sampling procedure to assess HIV seroconversion and associated factors among seronegative pregnant women attending ANC in Ethiopia, 2020.

From among the ten public health facilities in the study area, we randomly selected six public health facilities that provide routine antenatal care with comprehensive HIV counseling and testing. To determine the expected number of pregnant women in the June and July 2020 study period, we referred to antenatal care (ANC) follow-up data from June and July 2019. The total sample size was then proportionally distributed among the six selected public health facilities based on the number of HIV-negative pregnant women who attended their first antenatal screening. Then, the sampling fraction for each of the health facilities was determined by the probability proportional sampling method, and then a systematic random sampling technique was used, and kth women were included in the study (Figure 1).

### Data collection tools and procedures

A structured questionnaire based on the face-to-face interview technique and a review of participants' records were used to collect socio-demographic, clinical, and behavioral data. The questionnaires were adapted and piloted before the study. Data was collected by well-trained twelve midwives at the diploma level and three at the degree level of qualification in the selected healthcare facilities under strict supervision. Data were collected after the purpose of the study was explained to all eligible pregnant women and written and verbal informed consent was obtained from each study participant.

**Figure 2:**
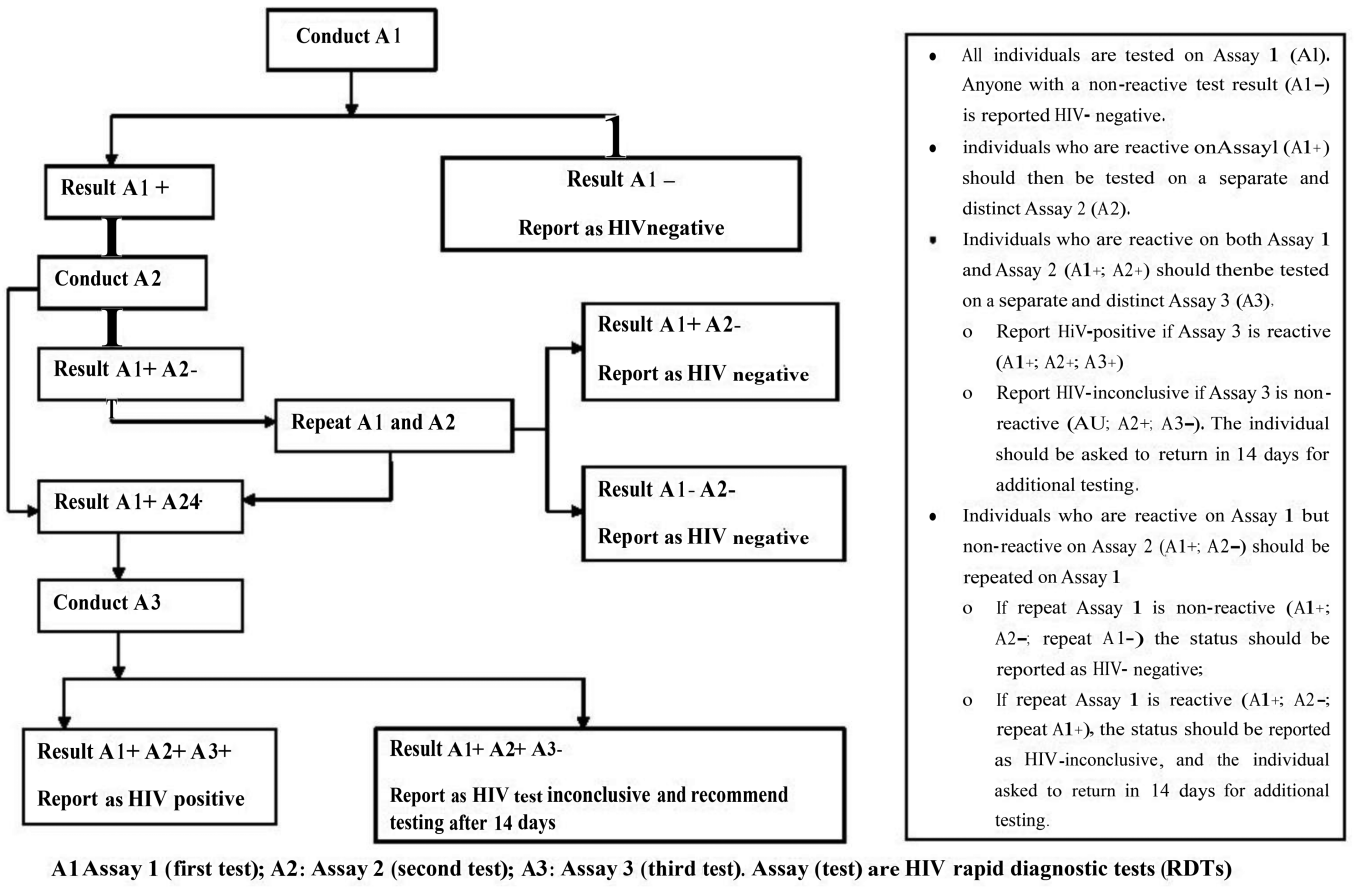
Recommended strategies for HIV testing in Ethiopia.

Moreover, provider-initiated voluntary testing and counseling (VCT) were performed for all study participants according to the national protocole (33) to determine the rate of HIV seroconversion following a negative test result 12 weeks after the initial test. The study used a WHO-prequalified kit for diagnosing HIV infection. Pretest counseling was given to all participants, followed by the collection of blood. The Chembio HIV 1/2 STAT-PAK Test, with a sensitivity of 99.5% and a specificity of 100%, was used to diagnose HIV infection. The HIV 1/2/0 Tri-line Human Immunodeficiency Virus Rapid Test Device, which has a sensitivity of 100%, and a specificity of 99.7%, was used as a second test for pregnant women who tested positive for HIV by the Chembio HIV 1/2 STAT-PAK test. The SD BIOLINE HIV-1/2/3.0 Rapid test was used as a confirmatory test with a sensitivity of 100%, and a specificity of 99.8% (34). Following retesting, post-test counseling was also employed (Figure 2).

### Data quality control

The questionnaire was first drafted in English and then translated into the local language and translated back into English to check for any inconsistencies. Data collectors and supervisors attended a two-day training session on the overall objective of the study and the content of the questionnaire administered by the principal investigator. Before the actual data collection, a pretest was then carried out on a 5% sample size in one of the health facilities outside the study area to ensure the validity of the instrument; the appropriate adjustment was then made. Throughout the data collection period, the data collection process was closely monitored daily.

### Operational definition of terms

#### Retest

HIV testing at least three months after an initial documented negative test result (30).

#### HIV seroconversion during pregnancy

Having an HIV-negative result at the initial HIV test and an HIV-positive result on a retest at least 12 weeks later (35).

#### Booking

The first documented antenatal care visit.

#### Knowledge of HIV infection

Was assessed using 12 items with “yes” or “no” questions. Participants who scored above the mean score were considered knowledgeable, and those who scored less than the mean score were considered not knowledgeable.

### Data processing and analysis

The data collected were entered into Epi data version 4.4.1 and exported and analyzed by SPSS version 25. Descriptive statistical analyses such as simple frequencies, measures of central tendency, and measures of variability were used to describe participants' characteristics. Then the information was presented using frequencies, summary measures, tables, and figures (charts). Beyond descriptive statistics, a bivariate analysis, Crude Odds Ratio (COR) with 95% CI, was used to see the association between each independent variable and the outcome variable using binary logistic regression. Independent variables with a *p*-value of <0.25 in the bivariate analysis were included in the multivariable analysis to detect independent factors of HIV seroconversion. An Adjusted Odds Ratio (AOR) with a 95% CI was estimated using multivariable logistic regression analysis to identify factors associated with HIV seroconversion. The level of statistical significance was defined as a *p*-value < 0.05.

### Ethical consideration

All methods were performed in accordance with relevant guidelines and regulations, from study design to analysis. Ethical approval for this study was obtained from the Research and Ethical Review Committee/Institutional Review Board, School of Public Health, College of Health Science of Addis Ababa University. Once approved, an official letter of cooperation was prepared for the relevant authorities and obtained from the Addis Ababa University School of Public Health. Permission to conduct the study was also obtained from the Kobo Town and Raya Kobo Woreda Health Bureau and the relevant health authorities. Then, after a clear explanation of the purpose, procedure, risks, and benefits of the study, and the importance of their participation, verbal informed consent was obtained from the illiterate and written consent from those who were literate. Parental consent was obtained from all pregnant women under 18 years of age. Participants were informed that they have the right to withdraw from the study at any time. Privacy and confidentiality were maintained for all study participants by coding results known only to caregivers. Moreover, the original data is kept in cabinets until the entire process is completed. All Pregnant women who were HIV positive during retesting were referred to a PMTCT clinic, and appropriate treatment was provided according to the Ethiopian National Guidelines for PMTCT by trained healthcare professionals at the relevant health facility.

## Results

### Socio-demographic and obstetrics characteristics of study participants

Of the 509 pregnant women recruited for the study, 494 were volunteers and participated in the study; the remaining 15(2.9%) women declined; this resulted in a response rate of 97.1%. Women who did not take part in the study did not want to be tested again because they believed they could not have been infected after testing negative in early pregnancy. The minimum and maximum age of the study participants was 15 and 45 years, respectively. The median age (IQR) of respondents was 30 (25–35) years and almost half of the respondents, 253 (51.2%), fell in the age group of 25–34 years. Most of them lived in urban areas 326 (66.0%) and were married 454 (91.9%). Most of the 359 (72.7%) respondents had ever attended school; of which, 157 (43.7%) and 89 (24.8) respondents have completed at least primary school and higher education, respectively. The median age of participant partners was 35 (IQR 30–40) years. The majority of 354 (71.7%) study participant partners attended school; of these, 91 (25.7%) have completed their higher education. Four hundred and two (81.4%) of the study participants had a lower perceived income. Most of the 300 participants (60.7%) had their first antenatal follow-up between 11 and 20 weeks of gestation. Approximately 417 (84.4%) pregnant women admitted that their pregnancy was planned (Table 1).

**Table 1:** Socio-demographic and obstetrics characteristics of the study participants (*n* = 494).

Variables	Frequency	Percent (%)
Age (completed years)		
15–24	104	21.1
25–34	253	51.2
≥35	137	27.7
Residence		
Urban	326	66.0
Rural	168	34.0
Marital status		
Single	34	6.90
Separated/divorced	6	1.20
Married	454	91.9
Religion		
Orthodox	381	77.1
Muslim	109	22.1
Other	4^a^	0.80
Ever attend school		
Yes	359	72.7
No	135	27.3
Educational status (*n* = 359)		
Higher education	89	24.8
Secondary education	113	31.5
Primary education	157	43.7
Occupational status		
Employed	89	18.0
Housewife	223	45.1
Farmer	110	22.3
Unemployed	52	10.5
Other^b^	20	4.00
Partner age		
15–24	43	8.7
25–34	180	36.4
≥35	271	54.9
Partner ever attends a school		
Yes	354	71.7
No	140	28.3
Partner educational status (*n* = 354)		
Higher education	91	25.7
Secondary school	108	30.5
Primary school	155	43.8
Partner occupational status (494)		
Employed	153	31.0
Merchant	78	15.8
Farmer	155	31.4
Unemployed	88	17.8
Other^c^	20	4.00
Household monthly income (average estimate)		
Lower-income	402	81.4
Low middle income	92	18.6
Gravidity		
1	146	29.6
2–3	211	42.7
≥4	137	27.7
Parity		
0	156	31.6
1–2	212	42.9
≥3	126	25.5
Gestational age at booking		
≥21 week	103	20.9
11–20 week	300	60.7
≤10 week	91	18.4
Status of pregnancy		
Planned	417	84.4
Unplanned	77	15.6

^a^ Protestant, catholic.

^b^ Student, merchant, daily laborer.

^c^ Daily laborer, driver, student.

### HIV infection knowledge characteristics

Regarding the participant's knowledge of HIV, 361 (73.1%) of the participants had relatively good knowledge of HIV. Almost all participants heard of HIV 492 (99.6%). Of these, 377 (76.6%) said they could reduce HIV infection just by having only one uninfected sex partner. More than half of the women, 312 (63.4%), believe that a person infected with HIV will always show signs and symptoms, and approximately two hundred and ninety-seven (60.4%) know that a person who appears healthy but is infected with HIV viruses can be transmitted to others. The majority of 336 (68.3%), 341 (69.3%), and 313 (63.6%) women are aware that HIV can be transmitted during pregnancy, childbirth, and breastfeeding, respectively (Table 2).

**Table 2:** HIV infection knowledge of study participants (*n* = 494).

Variables	Frequency	Percent (%)
Ever heard of HIV		
Yes	492	99.6
No	2	0.40
Have only one sexual partner reduced HIV risk (*n* = 492)		
Yes	377	76.6
No	115	23.4
HIV transmitted by mosquito bite (*n* = 492)		
Yes	193	39.2
No	299	60.8
Condom utilization reduces HIV risk (*n* = 492)		
Yes	355	72.2
No	137	27.8
HIV transmitted by sharing food (*n* = 492)		
Yes	88	17.9
No	404	82.1
HIV transmitted by witchcraft (*n* = 492)		
Yes	178	36.2
No	314	63.8
HIV-infected people always show signs (*n* = 492)		
Yes	312	63.4
No	180	36.6
Healthy looking HIV positive person can be infectious (*n* = 492)		
Yes	297	60.4
No	195	39.6
HIV transmitted during pregnancy (*n* = 492)		
Yes	336	68.3
No	156	31.7
HIV transmitted during delivery (*n* = 492)		
Yes	341	69.3
No	151	30.7
HIV transmitted by breastfeeding (*n* = 492)		
Yes	313	63.6
No	179	36.4
There is a special drug to reduce vertical transmission of HIV(*n* = 487)		
Yes	205	42.1
No	282	57.9

HIV, human immunodeficiency virus.

### Clinical characteristics

From a total of 494 pregnant women, twenty-six (5.3%) were diagnosed with sexually transmitted infections; of these, fifteen (3%) were women diagnosed with malodorous vaginal discharge, and eleven (2.2%) with a genital ulcer or sore. Out of the study participants, 29 (5.9%) partners had sexually transmitted infections; of these, eighteen of them (3.6%) had abnormal urethral discharge, and eleven (2.2%) had genital ulcers or sore (Table 3).

**Table 3:** Clinical characteristics of the study participants (*n* = 494).

Variables	Frequency	Percent (%)
Ever diagnosed with STI		
Yes	23	4.70
No	471	95.3
Had a bad-smelling vaginal discharge		
Yes	15	3.00
No	479	97.0
Had a genital ulcer or sore		
Yes	11	2.20
No	483	97.8
Has had an STI		
Yes	26	5.30
No	468	94.7
Partner has ever been diagnosed with STI		
Yes	21	4.30
No	473	95.7
Partner had urethral discharge		
Yes	18	3.60
No	476	96.4
Partner had a genital ulcer or sore		
Yes	11	2.20
No	483	97.8
Partner had STI		
Yes	29	5.90
No	465	94.1

STI, sexually transmitted infections.

### Sexual and behavioral characteristics

A total of 346 (70.0%) pregnant women had consumed alcohol. The majority of participants 147 (42.5%) drank alcohol less than once a week, and almost a quarter (24.8%) of participants drank alcohol almost every day. More than three-quarters of the participants, 375 (82.6%), lived with their partners, and 67 (13.6%) of the participants traveled frequently for work. Only 94 (19%) of participants are fully aware of their partners' HIV status; of those who do know, only 1 (1.1%) of the participant's partners had HIV. The median age at first intercourse was 16 years (IQR 15–18). Only 42(8.5%) of study participants used a condom the last time they had sex. Also, 4 (0.8%) participants had sex with someone other than their partners. Thirty (6.1%) participants suspect their partner has another sexual partner, and 10 (2.0%) participant partners have another wife. 49 (9.9%), 44 (8.9%), and 31 (6.3%) of study participants experienced physical, emotional, and sexual abuse from their partners, respectively (Table 4).

**Table 4:** Sexual and behavioral characteristics of the study participants (*n* = 494).

Variables	Frequency	Percent (%)
Ever drink alcohol		
Yes	346	70.0
No	148	30.0
How often you drink alcohol (*n* = 346)		
Almost everyday	86	24.8
At least once a week	113	32.7
Less than once a week	147	42.5
Partner living with her or not (*n* = 454)		
Living with her	375	82.6
Staying elsewhere	79	17.4
Partner traveled for work frequently		
Yes	67	13.6
No	427	86.4
New partner in the last three months		
Yes	5	1.00
No	489	99.0
Duration of relationship		
≤1 year	62	12.6
>1 year	432	87.4
Partner ever drinks alcohol		
Yes	446	90.3
No	48	9.70
How often did your partner take alcohol (*n* = 446)		
Almost everyday	230	51.6
At least once a week	157	35.2
Less than once a week	59	13.2
Close knowledge about partner's HIV status		
Yes	94	19.0
No	400	81.0
Partner HIV status (*n* = 94)		
HIV positive	1	1.10
HIV negative	93	98.9
Age at first sex		
≤15	149	30.2
16–17	208	42.1
≥18	137	27.7
Time of last sexual intercourse		
Days ago	77	15.6
Weeks ago	128	25.9
Months ago	289	58.5
Participants used a condom during their last sexual intercourse		
Yes	42	8.50
No	452	91.5
Had sex with other than their partner		
Yes	4	0.80
No	490	99.2
Partner has other wives		
Yes	10	2.00
No	484	98.0
Suspect their partner with another sexual partner		
Yes	30	6.10
No	464	93.9
Emotionally abused		
Yes	49	9.90
No	445	90.1
Physically abused		
Yes	44	8.90
No	450	91.1
Sexually abused		
Yes	31	6.30
No	463	93.7

HIV, human immunodeficiency virus.

### HIV status of the pregnant women after re-testing

From a total of 494 pregnant women who were screened and reported negative for HIV at first ANC, six (1.2%) were HIV seropositive during retesting.

### Factors associated with HIV seroconversion

On bivariate logistic regression model, marital status, gestational age at booking, pregnancy status, participants with a reported history of STI, partners of participants with a reported history of STI, partners of participants with a reported history of frequent business trips, length of the relationship, participants who suspected their partner of having other sexual partners, and participants who had been sexually abused by their partners were eligible for the final multivariable logistic regression model.

After controlling for the effects of other predictor variables, the multivariable logistic regression analysis showed that participants with a reported history of STI, partners with a reported history of frequent business travel, and participants who were sexually abused by their partner were significantly associated with HIV seroconversion (*p*-value < 0.05). Pregnant women with a reported history of STI are more likely to have HIV seroconversion [AOR = 7.98; 95% CI (1.21, 52.82)] compared to pregnant women with no history of STI. The odds of participants' partners' reported travel history for work frequently were significantly higher than those partners who did not report travel history [AOR = 6.00; 95% CI (1.09, 32.99)]. Sexually abused pregnant women have 7.8 higher odds [AOR = 7.82; 95% CI (1.19, 51.24)] of HIV seroconversion than those with no reported claim of partner sexual abuse (Table 5).

**Table 5:** Factors associated with HIV seroconversion (*n* = 494).

Variable	HIV seroconversion			
	No (%)	Yes (%)	COR (95%CI)	AOR (95%CI)
Marital status				
Married	450 (99.1)	4 (0.9)	1	1
Separated/divorced	6 (100.0)	0 (0.0)	–	–
Single	32 (94.1)	2 (5.9)	7.03 (1.24, 39.85)	2.78 (0.31, 25.21)
GA at booking				
≤10 week	89 (97.8)	2 (2.2)	1	1
11–20 week	298 (99.3)	2 (0.7)	0.30 (0.04, 2.15)	0.25 (0.03, 2.04)
≥21 week	101 (98.1)	2 (1.9)	0.88 (0.12, 6.39)	0.48 (0.04, 5.30)
Status of pregnancy				
Planned	413 (99.0)	4 (1.0)	1	1
Unplanned	75 (97.4)	2 (2.6)	2.75 (0.49, 15.30)	4.26 (0.61, 29.86)
Had STI				
No	464 (99.1)	4 (0.9)	1	1
Yes	24 (92.3)	2 (7.7)	9.67 (1.69, 55.42)	7.98 (1.21, 52.82)*
Partner had STI				
No	461 (99.1)	4 (0.9)	1	1
Yes	27 (93.1)	2 (6.9)	8.54 (1.50, 48.70)	5.05 (0.62, 40.84)
Partner traveled for work frequently				
No	424 (99.3)	3 (0.7)	1	1
Yes	64 (95.5)	3 (4.5)	6.62 (1.31, 33.53)	6.00 (1.09, 32.99)*
Duration of relationship				
>1year	428 (99.1)	4 (0.9)	1	1
≤1 year	60 (96.8)	2 (3.2)	3.57 (0.64, 19.89)	0.86 (0.03, 22.19)
Suspect partners with another sexual partner				
No	460 (99.1)	4 (0.9)	1	1
Yes	28 (93.3)	2 (6.7)	8.21 (1.44, 46.79)	1.78 (0.16, 20.45)
Sexually abused				
No	459 (99.1)	4 (0.9)	1	1
Yes	29(93.5)	2 (6.5)	7.91(1.39, 45.01)	7.82(1.19, 51.24)*

STI, sexually transmitted infections; COR, crude odds ratio; AOR, adjusted odds ratio; CI, confidence interval; 1, reference category.

* *P-*value < 0.05

## Discussion

This study documented an HIV seroprevalence rate of 1.2% in antenatal attendees' women who were seronegative early in pregnancy. The seroconversion rate in this study was somewhat low compared to a study in other countries like 2% in Tanzania (36), 2.5% in Nigeria (37), 2.6% in Kenya (18), 3% in South Africa (29), 4% in Swaziland (27), 4.3% in Mozambique (38), and 17.7% in Zimbabwe (39). Nonetheless, the findings show that HIV seroconversion remains a public health problem for pregnant women. The magnitude varies from country to country, this may be because different countries have different HIV intervention strategies and use different sample sizes. The low seroconversion rate in this study is due to differences in socio-demographic characteristics as well as the fact that other studies were conducted in areas of high HIV prevalence.

Low HIV seroconversion rates of 0.68% and 0.25% were also reported in South Africa and Nigeria, respectively (14, 40). It could be since the pregnant women in these studies received antenatal care in early pregnancy, which helped them receive health education for risky sexual behavior, which in turn protects against sexually transmitted infections, including HIV.

In this study, single pregnant women (5.9%) were more likely to seroconvert than married women (0.9%). This is consistent with a study in South Africa (29) which found that single pregnant women were more likely to seroconvert during pregnancy. This may be because single women may be predisposed to other sexual relationships, increasing the risk of HIV infection.

Pregnant women with a reported history of sexually transmitted infections were significantly associated with HIV seroconversion in this study (*p* = 0.031). A study in Tanzania (13) also found that pregnant women with STIs were significantly associated with HIV seroconversion (*p* < 0.001). The study also found that partners of pregnant women with STIs were seven times more likely to seroconvert than partners without STIs. The very fact could partly explain this is that STIs will increase HIV susceptibility by disrupting the epithelial surface of the genital tract, thus providing a straightforward port of entry by activating HIV susceptibility inflammatory cells; the inflammation increases HIV shedding in the genital tract, hence the HIV infectiousness (41).

This study showed that pregnant partners of frequent travelers were significantly associated with HIV seroconversion (*p* = 0.039). This is also supported by a study in Tanzania (42); showing that pregnant women with a mobile partner were significantly associated with HIV infection (*p* = 0.003). Another author, Mbena H et al., also supported this study and showed that pregnant women with a mobile partner were more likely to seroconvert, though this was not statistically significant (13). This can be partly explained by the fact that men who have to travel frequently for work are often suspected of riskier sexual behaviors, including drinking too much alcohol, having another sex partner, and having sex with prostitutes, putting them and their partners at risk for HIV infection. On the other hand, it can be assumed that women with mobile partners are at risk of having sex outside of marriage since they are free, lonely, or have financial difficulties (39, 42).

Surprisingly, only 19% of HIV-negative pregnant women know their partner's HIV status, while none of the seroconverted pregnant women know their partner's HIV status. This figure is low compared to a study in South Africa which showed that 52% of seronegative and 66% of seroconverting pregnant women know their partner's HIV status (43). This can be explained by the fact that partners' HIV testing policies in Ethiopia are not well implemented or integrated into ANC services (44). It also means that pregnant women's partners do not commit to attending antenatal care. This is supported by Caroline De Schacht et al.; who showed fear of discrimination and stigma was the main barrier to male HIV testing (38). However, most studies in Ethiopia and elsewhere show that male partner involvement in maternal and child health services is associated with increased uptake of maternal health services, such as antenatal care (ANC), the likelihood of having a facility-based delivery, use of contraceptives, and reducing mother-to-child transmission of HIV (MTCT) (45). Studies have also shown that couples who receive counseling together are more likely to accept HIV tests (46), and are more likely to adhere to antiretroviral therapy (47, 48). According to Aluisio et al., male partners participating in maternal healthcare have a lower chance of contracting HIV as newborns of HIV-positive mothers and have higher HIV-free survival rates (49).

There was only one serodiscordant couple in this study, and she tested negative upon retesting. Since the index man may have been treated with ARVs and HIV is well controlled to an undetectable viral load, along with other preventive measures such as using barriers and practicing safe sex, she may not have contracted the virus. Therefore, it is helpful to use various HIV prevention strategies, such as pre-exposure prophylaxis (PrEP), antiretroviral therapy, and condoms which can assist both partners in managing and maintaining their health and is more effective than using just one. Furthermore, the absence of sexually transmitted diseases like bacterial vaginosis and herpes simplex can also reduce the risk of transmission.

In the findings, only 8.5% of the participants reported using condoms, while none of the seroconverted pregnant women used condoms during pregnancy. Moodley et al. (50) also reported that only 2.8% of seroconverted pregnant women use condoms during pregnancy. Both findings suggest that pregnant women who do not use condoms are more likely to seroconvert. It could be because couples usually do not use condoms: and if their partner has another sex partner, the risk of contracting STIs increases, making them more susceptible to HIV infection.

This study revealed that pregnant women who suspected their partner had another sexual partner were seven times more likely to seroconvert than their counterparts. Correspondingly, a study in Nigeria also found that pregnant women who suspected their partner had another sexual partner were three times more likely to seroconvert than their counterparts (51). The most likely scenario is that many married women remain sexually abstinent during pregnancy, during which time their partners are at risk of contracting STIs, including HIV, through other sexual relationships (39).

This study showed a significant association between sexual abuse during pregnancy and HIV seroconversion (*p* = 0.032). Likewise, a study in Zimbabwe found that pregnant women who reported sexual abuse were significantly associated with HIV seroconversion (52). It could partly be due to the very fact that women who have been sexually abused may have forced sexual intercourse without a condom or be unable to negotiate condom use with their partner, increasing the risk of HIV infection. Another study found that men who engaged in violence against their partner were more likely to engage in high-risk sexual behaviors, such as multiple sex partners, sex without condoms, excessive drinking, and intravenous drug abuse than non-violent men (53, 54). Biological mechanisms are also thought to play a role in indirectly increasing the risk of HIV and other sexually transmitted infections resulting from genital trauma (55).

A limitation of this study is that the test we used to diagnose HIV was a rapid serological test that could not detect the infection in the window period. This reduces the actual seroconversion rate. Furthermore, the study does not tell us whether the seroconversion was due to a new infection or following the window period due to the cross-sectional nature of the design. With a due balance of strengths and limitations, it is reasonable to believe that the study provides valid baseline information about HIV seroconversion among pregnant women.

## Conclusions

Although the percentage of women who sero-converted was quite low, we demonstrate that there is a subset of pregnant women who are at risk of sero-conversion during pregnancy. Therefore, this study highlights that testing HIV-negative pregnant women in later stages of pregnancy can identify women who have seroconverted, thereby enabling the early use of Anti-Retroviral Treatment (ART) to prevent mother-to-child transmission of HIV infection, particularly in situations where the possibility of seroconversion or new infection cannot be clearly excluded. Based on the results of this study, we recommended that all healthcare professionals working in maternal and child health clinics repeat testing of HIV-negative pregnant women in late pregnancy to identify seroconverted pregnant women. The finding also underscores the need for couples to seek advice on safer sex practices and to actively investigate and treat sexually transmitted infections during pregnancy to prevent HIV infection. Studies using antigen-based tests are needed to determine whether seroconversion is due to recent illness or true seroconversion after the window period. Furthermore, expanded and large-scale studies need to be conducted to understand the extent and drivers of HIV seroconversion during pregnancy at different levels.

## Data Availability

The original contributions presented in the study are included in the article/**Supplementary Material**, further inquiries can be directed to the corresponding author.

## Supplementary material

Supplementary S1 File English version questionnaire. (PDF)

Supplementary S2 File Amharic version questionnaire. (PDF)

Supplementary S3 Data The raw data supports the finding of this article. (SAV)
