# Supplementary material for: HIV seroconversion and associated factors among seronegative pregnant women attending ANC in Ethiopia: an institution-based cross-sectional study
Source: Front Reprod Health. 2024 Apr 10;6:1246734. doi: 10.3389/frph.2024.1246734 (PMC11039893; doi:10.3389/frph.2024.1246734)
Supplement: Supplementary S1 — File English version questionnaire. (PDF) [file Datasheet1.pdf]

Annex 4: English version questionnaire

Questionnaire ID____________________

Name of the health facility____________________

Date of interview____________________

Part 1: General background information (socio demographic characteristics)

No	Questions	Coding categories	Remark
101	How old are you?	Age in completed years______	
102	Your usual residence?	Urban…………....…......…1<br>Rural……………………....2	
103	What is your marital status now?	Never married……………..1<br>Married……………………2<br>Widowed………….............3<br>Divorced…………………..4<br>Separated………….............5	
104	What is your religion?	Orthodox…………….……1<br>Muslim ……………...........2<br>Protestant….………….…...3<br>Catholic …………............4<br>Traditional…..…..….............5<br>Other……………….……..88	
105	Have you ever attended school?	Yes ……………………......1<br>No ……………………............2→	107
106	What is the highest level of school (education) you have attended?	Primary school ……………1<br>Secondary school ………...2<br>Technical/Vocational....…..3<br>Higher education. …………4	
107	What is your main occupation?	Government employee……1<br>Private employee ………...2<br>Merchant…………………....3<br>House wife……………..…....4<br>Farmer……………….………5<br>Daily laborer……….…….....6<br>Unemployed…..…………......7<br>Other…………….…………88	
108	How old is your husband/partner?	Age in completed Years ____	
109	Did your husband/partner ever attend school?	Yes ………..……...……1<br>No ………..………….......2→	111

110	What was the highest level of school (education) he had attended?	Primary school …………..1<br>Secondary school…......…...2<br>Technical/Vocational......….3<br>Higher education ….……..4<br>Don't know………………88	
111	What is your husband/partner's main occupation?	Government employee…….1<br>Private employee …………2<br>Merchant…………………..3<br>Farmer……………….……4<br>Daily laborer……………......5<br>Unemployed……………......6<br>Other………...…………88	
112	Household's average monthly income(Birr)	Birr ________	

Part 2: Obstetric related questions

201	During your life, how many times have you become pregnant including the current pregnancy (including a pregnancy that was miscarried/ aborted, or ended in a stillbirth)?<br>(verify her answer from the card where appropriate)	Times________	
202	During your life, how many times have you given live birth? (I mean, to a child who ever breathed or cried or showed other signs of life-even if he or she lived only a few minutes or hours)<br>(verify her answer from the card where appropriate)	Times________	
203	How many weeks of pregnant were you when you first received antenatal care for this pregnancy?<br>(verification from ANC card)	Weeks________	
204	Women can sometimes get pregnant without planning or needing to get pregnant; Did you plan this pregnancy?	Yes………………………1<br>No……………………….2<br>Uncertain………………88	

Part 3: MTCT of HIV knowledge related questions

301	Have you ever heard of HIV?	Yes…………….…..……1<br>No……………….……..2 →	Part 4
302	Can people reduce their chance of getting HIV by having just one uninfected sex partner who has no other sex partners?	Yes……………………..1<br>No………………………2<br>Don't know……………..88	
303	Can people get HIV from a mosquito bite?	Yes……………………..1<br>No……………………...2<br>Don't know…………….88	
304	Can people reduce their chance of getting HIV by using a condom every time they have sex?	Yes……………………..1<br>No……………………...2<br>Don't know………….....88	
305	Can people get HIV by sharing food with a person who has HIV?	Yes…………………….1<br>No……………………..2<br>Don't know…………….88	
306	Can people get HIV because of witchcraft or other supernatural means?	Yes………………….....1<br>No……………………..2<br>Don't know……………88	
307	Do HIV-infected people always show signs?	No (Can look healthy)…...1<br>Yes (Always show signs)...2<br>Don't know…………..…88	
308	Are healthy-looking infected men infectious?	Yes…………………….1<br>No……………………..2<br>Don't know……………88	
309	Can HIV be transmitted from a mother to her baby:<br>a) During pregnancy?<br>b) During delivery?<br>c) By breastfeeding?	YES NO DK<br>a)……………1 2 88<br>b)…………....1 2 88<br>c)……………1 2 88	
CHECK 309	AT LEAST ONE YES ☐ ↓	OTHER ☐ →	Part 4
310	Are there any special drugs that can give to a woman infected with HIV to reduce the risk of transmission to the baby?	Yes……………………..1<br>No……………………...2<br>Don't know…………….88	

Part 4: Clinical related questions

401	Have you ever been diagnosed with a sexually transmitted disease?	Yes……………………..1<br>No……………………...2<br>Don't know……………88	

402	Sometimes women experience a bad-smelling abnormal genital discharge. Have you had a bad-smelling abnormal genital discharge?	Yes……………………1<br>No……………………..2<br>Don't know……………88	
403	Sometimes women have a genital sore or ulcer. Have you had a genital sore or ulcer?	Yes……………………1<br>No……………………..2<br>Don't know……………88	
404	Check for 401, 402, and 403,<br>Has had an infection?	Yes……………………1<br>No……………………..2<br>Don't know……………88	
405	Has your husband / partner been diagnosed with sexually transmitted disease?	Yes……………………1<br>No……………………..2<br>Don't know…………..88	
406	Sometimes men experience an abnormal discharge from their penis. Has your husband/partnerhad an abnormal discharge from his penis?	Yes……………………1<br>No……………………..2<br>Don't know…………....88	
407	Sometimes men have a sore or ulcer near their penis. Have your husband/partner had a sore or ulcer on or near his penis?	Yes……………………1<br>No………..…………...2<br>Don't know……………88	
408	Check 405, 406, and 407,<br>Has a husband/partner had an infection?	Yes……………………1<br>No………..…………...2<br>Don't know……………88	

Part 5: Sexual and behavioral related questions

501	Have you ever taken a drink that contains alcohol(Tella/Tegii/Areke/Beer/Wine, etc...)?	Yes……………………1<br>No……………………2 →	504
502	How often did you take a drink that contains alcohol?	Almost every day……......1<br>At least once a week……..2<br>Less than once a week…..3<br>At least once a month…....4	
503	Is your husband/partner living with you now or is he staying elsewhere? (married only)	Living with her…………1<br>Staying elsewhere……....2	
504	Does your husband/partner travel for work frequently?	Yes…….……………….1<br>No………..…………..…2	
505	Do you have a new husband/partner in	Yes………..……………...1	

	the last three months?	No…………..……………2	
506	How long have you been together with your husband/partner?	___________	
507	Has your husband/partner ever taken a drink that contains alcohol (Tella/Tegii/Areke/Beer/Wine, etc...)?	Yes……………………1<br>No…………………….2<br>Don't know …………88	→ 511
508	How often did your husband/partner take a drink that contains alcohol?	Almost every day………..1<br>At least once a week……..2<br>Less than once a week…....3<br>At least once a month……4	
509	Do you have close knowledge about the HIV status of your husband/partner?	Yes…………….……...1<br>No……..…………………..2	→ 513
510	What is your husband/partner's HIV status?	Has HIV ………………1<br>No HIV………….……...2	
511	Now I would like to ask some questions about sexual activity in order to gain a better understanding of some important life issues. Let me assure you again that your answers are completely confidential and will not be told to anyone. If we should come to any question that you don't want to answer, just let me know and we will go to the next question. How old were you when you had sexual intercourse for the very first time?	Age in years _______	
512	When was the last time you had sexual intercourse?	Days ago__________<br>Weeks ago_________<br>Months ago________	
513	The last time you had sexual intercourse, was a condom used?	Yes…………………......1<br>No………………………2<br>Uncertain……………….3	
514	Apart from your husband/partner, have you had sexual intercourse with any other person?	Yes………………...……1<br>No……………..…...……2	→ 518
515	In total, with how manydifferent people have you had sexual intercourse?	People________<br>Don't know……………88	

516	Does your husband/partner have other wives or does he lives with other women as if married?	Yes……………………….1<br>No……………………….2<br>Don't know…………….88	
517	Do you suspect your husband/partner has another sexual partner?	Yes……………………….1<br>No……………………......2<br>Don't know……………..88	
518	Does your husband/partner abuse you emotionally (like insults, talkdown to, threaten to harm, scream, curse you, and make you feel bad about yourself)?	Never……………….…..…..1<br>Sometimes…………..…..2<br>Often…………………......3<br>Almost always…………..4	
519	Does your husband/partner abuse you physically (like a hit, slapped,kicked, push you)?	Never……………..…….1<br>Sometimes……….………2<br>Often………..…………3<br>Almost always.…………..4	
520	Does your husband/partner abuseyou sexually (like forced to have sexual intercourse when you did not want to)?	Never…………………......1<br>Sometimes……………....2<br>Often…………………......3<br>Almost always…………...4	

Part 6: HIV status of the women

601	The HIV status of the women after retesting?	HIV negative…….………1<br>HIV positive………..…....2	

Thank you for your time

Name of interviewer___________________________Signature__________

Name of supervisor____________________________Signature__________
