# Supplementary material for: HIV seroconversion and associated factors among seronegative pregnant women attending ANC in Ethiopia: an institution-based cross-sectional study
Source: Front Reprod Health. 2024 Apr 10;6:1246734. doi: 10.3389/frph.2024.1246734 (PMC11039893; doi:10.3389/frph.2024.1246734)
Supplement: Supplementary S2 — File Amharic version questionnaire. (PDF) [file Datasheet2.pdf]

አባሪ 3፡- የአማርኛ መጠይቅ

የጤናተቋሙ ስም___________________________

የመጠይቁ መለያ____________________________

መጠይቁ የተሞላበት ቀን______________________

ክፍል አንድ፡-አጠቃላይ የማህበራዊ እና ስነ ህዝብ ተዛማች ጥያቄዎች

ቁጥር	ጥያቄዎች	የምልክት ምድብ	አስተያየት
101	እድሜዎ ስንት ነው?	_______	
102	ቋሚ የመኖሪያ አድራሻዎ ?	ከተማ..1<br>ገጠር..2	
103	አሁን ያሉበት የጋብቻ ሁኔታዎ ምንድን ነው?	ፈፅሞ ያላገባች...............................1<br>ያገባች..2<br>ባልዋ የሞተባት ....3<br>የተፋታች.......................................4<br>የተለያየች 5	
104	ሃይማኖትዎ ምንድን ነዉ?	ኦርቶዶክስ ክርስቲያን......................1<br>ሙስሊም.......................................2<br>ፕሮቴስታንት.................................3<br>ካቶሊክ..4<br>ባህላዊ...5<br>ሌላ...88	
105	ትምህርት ተምረው ያዉቃሉ?	አወ...1<br>አላዉቅም..................................2 ⟶	 107
106	እርስዎ የጨረሱት/ የደረሱበት ከፍተኛ የትምህርት ደረጃ?	የመጀመሪያ ደረጃ ....1<br>ሁለተኛ ደረጃ ....2<br>ቴክኒክ እና ሙያ............................3<br>ከፍተኛ ትምህርት 4	
107	ዋና/መደበኛ ስራዎ ምንድነዉ?	የመንግስት ተቀጣሪ......................1<br>የግል ተቀጣሪ...............................2<br>ነጋዴ...3<br>የቤት እመቤት.............................4<br>አርሶ አደር...................................5<br>የቀን ሰራተኛ................................6<br>ስራ የሌላት...................................7<br>ሌላ...88	

108	የባለቤትሽ/ የፍቅር ጓደኛሽ እድሜ ስንት ነው?	________	
109	ባለቤትሽ/ የፍቅር ጓደኛሽ ትምህርት ተምሯል?	አወ..1<br>አልተማረም..................................2 →	 111
110	ባለቤትሽ/ የፍቅር ጓደኛሽ የጨረሱት/ የደረሱበት ከፍተኛ የት/ት ደረጃ?	የመጀመሪያ ደረጃ.........................1<br>ሁለተኛ ደረጃ................................2<br>ቴክኒክ እና ሙያ...........................3<br>ከፍተኛ ትምህርት..........................4<br>አላውቅም....................................88	
111	የባለቤትሽ/ የፍቅር ጓደኛሽ ዋና/መደበኛ ስራው ምንድን ነው?	የመንግስት ተቀጣሪ.......................1<br>የግል ተቀጣሪ................................2<br>ነጋዴ...3<br>አርሶ አደር....................................4<br>የቀን ሰራተኛ.................................5<br>ስራ የሌለዉ....................................6<br>ሌላ..88	
112	የቤተሰብ አማካይ ወርሃዊ ገቢ (በብር)?	________	

ክፍል ሁለት፤- ስነ ተዋልዶ ጋር ተዛማችነትያላቸዉ ጥያቄዎች

201	የአሁኑን ዕርግዝና ጨምሮ በሕይወትዎ ውስጥ ስንት ጊዜ ነፍሰጡር ሆነዋል?<br>(ፅንሱ የተጨናገፈ/ የወረደ/ሞቶ የተወለደን ይጨምራል)<br>(መልሱ ተገቢ ከሆነ ከካርዱ ላይ ያረጋግጡ)	________	
202	በህይወትዎ ውስጥ ስንት ጊዜ በሕይወት ያለ ልጅ ወልደዋል? (ማለትም ሲወለድ እስትንፋስ የነበረው/ ያለቀሰ /ሌሎች የህይወት ምልክቶች የነበሩት ምንም እንኳን ከተወለደ/ች ጥቂት ደቂቃ /ሰዓት ብቻ በሕይወት ቢኖርም/ ብትኖርም)<br>(መልሱ ተገቢ ከሆነ ከካርዱ ላይ ያረጋግጡ)	________	
203	የመጀመሪያውን የቅድመ ወሊድ ክትትል ስታደርጊ የስንት ሳምንታት ነፍሰ ጡር ነበርሽ? (ማረጋገጫ ከ ANC ካርድ)	________	
204	ሴቶች አንዳንድ ጊዜ እቅድ ሳያወጡ ወይንም ሳይፈልጉ እርጉዝ ሊሆኑ ይችላሉ ፣ እርስዎስ ይሄን እርግዝና አቅደው ነው ያረገዙት?	አዎ .... 1<br>አይደለም ....2<br>እርግጠኛ አይደለሁም 88	

ክፍል ሦስት፡- ኤች አይ ቪ ዕውቀት ጋር ተዛማችነትያላቸዉ ጥያቄዎች

301	ስለ ኤች አይ ቪ ሰምተዉ ያዉቃሉ?	አወ...1<br>አላዉቅም..................................2 →	ክፍል አራት
302	ሰወች ሌላ የወሲብ ጉዋደኛ ከሌለዉ እና ከ ኤች አይ ቪ ነፃ ከሆነ ጓደኛ ጋር ብቻ በመሆን በ ኤች አይ ቪ የመያዝ እድላቸዉን ሊቀንሱ ይችላሉ?	አወ...1<br>አይችሉም..................................2<br>አላዉቅም.................................88	
303	ሰወች በትንኝ ንክሻ ኤች አይ ቪ ሊያዙ ይችላሉ?	አወ...1<br>አይያዙም..................................2<br>አላዉቅም.................................88	
304	ሰወች የግብረ-ስጋ ግኑኝነት በሚፈሙበት ወቅት ኮንዶም ቢጠቀሙ በኤች አይ ቪ የመያዝ እድላቸዉን ሊቀንሱ ይችላሉ?	አወ..1<br>አይችሉም.................................2<br>አላዉቅም................................88	
305	ሰወች ኤች አይ ቪ ካለበት ሰዉ ጋር ምግብ ቢመገቡ ኤች አይ ቪ ሊያዙ ይችላሉ?	አወ..1<br>አይያዙም.................................2<br>አላዉቅም................................88	
306	ሰዎች በ ጥንቆላ ወይንም መለኮታዊ በሆኑ ምክኒያቶች ኤች አይ ቪ ሊያዙ ይችላሉ	አወ..1<br>አይያዙም................................2<br>አላዉቅም................................88	
307	በ ኤች አይ ቪ የተያዙ ሰወች ሁል ጊዜ የህመም ምልክት ያሳያሉ?	አያሳዩም..................................1<br>ያሳያሉ....................................2<br>አላዉቅም...............................88	
308	ጤናማ የሚመስል ግን ኤች አይ ቪ ያለበት ሰው በሽታውን ሊያስተላልፍ ይችላል?	አወ..1<br>አይችልም.................................2<br>አላዉቅም...............................88	
309	ኤች አይ ቪ ከ እናት ወደ ልጅዋ ሊተላለፍ ይችላል?<br>ሀ) በዕርግዝና ወቅት<br>ለ) በወሊድ ጊዜ<br>ሐ) ጡት በማጥባት	አወ አይችልም አላዉቅም<br>ሀ).............1 2 88<br>ለ).............1 2 88<br>ሐ).............1 2 88	
309 ኝን ያረጋግጡ ቢያንስ አንድ አወ<br>ካለ ↓		ከሌለ →	ክፍል አራት
310	ኤች አይ ቪ ከእናት ወደ ልጅ እንዳይተላለፍ የሚያግዝ ኤች አይ ቪ ለተያዙ ሴቶች ሊሰጥ የሚችል ልዩ መድሃኒት አለ	አወ...1<br>የለም...2<br>አላዉቅም..................................88	

ክፍል አራት፤- ከህክምና ጋር ተዛማችነት ያላቸዉ ጥያቄዎች

401	በጾታዊ ግንኙነት አማካኝነት በሚመጣ በሽታ ተይዘዉ ያዉቃሉ?	አዎ ....1<br>አልተያዝኩም..................................2<br>አላውቅም....................................88	
402	አንዳንድ ጊዜ ሴቶች ያልተለመደ ሽታ ያለው የብልት ፈሳሽ ያጋጥማቸዋል፡ አርስዎስ ያልተለመደ ሽታ ያለው የብልት ፈሳሽ አጋጥመዎት ያዉቃል?	አወ..1<br>አላጋጠምኝም...................................2<br>አላውቅም....................................88	
403	አንዳንድ ጊዜ ሴቶች የብልት ቁስለት ያጋጥማቸዋል፡ አርስዎስ የብልት ቁስለት አጋጥመዎት ያዉቃል?	አወ..1<br>አላጋጠምኝም.................................2<br>አላውቅም....................................88	
404	401ን፣402ን፣አና 403ን ያረጋግጡ እንፈክሽኑ ነበረባት?	አወ..1<br>አልነበረባትም..................................2<br>አታውቅም....................................88	
405	ባለቤትዎ/የፍቅር ጓደኛዎ በጾታዊ ግንኙነት በሚመጣ በሽታ ተይዞ ያዉቃል?	አወ..1<br>አልተያዘም.....................................2<br>አላውቅም....................................88	
406	አንዳንድ ጊዜ ከወንዶች ብልት ዉስጥ ያልተለመደ ፈሳሽ ይወጣል፡ ከባለቤትሽ/ከፍቅር ጓደኛሽ ብልት ውስጥ ያልተለመደ ፈሳሽ ይወጣ ነበር?	አወ..1<br>አይወጣም......................................2<br>አላዉቅም.....................................88	
407	አንዳንድ ጊዜ ከወንዶች ብልት አካባቢ ቁስል ይኖራል ፡ ከባለቤትሽ/ከፍቅር ጓደኛሽ ብልት አካባቢ ቁስለት ነበር?	አወ..1<br>አልነበረም......................................2<br>አላዉቅም....................................88	
408	405ን፡ 406ን፡ እና 407ን ያረጋግጡ እንፈክሽኑ ነበረበት?	አወ..1<br>አልነበረበትም.................................2<br>አታዉቅም....................................88	

አምስት፣ ወሲባዊ እና ባህሪ ጋር ተዛማጅነት ያላቸው ጥያቄዎች

501	አልኮል የያዘ መጠጥ ጠጥተው ያውቃሉ? (ለምሳሌ ጠላ ፣ አረቂ፣ ጠጅ፣ ቢራ ፣ ወይን ወዘተ)	አወ..1<br>አላዉቅም......................................2→	 503
502	በ ምን ያህል ጊዜ ውስጥ ነው አልኮል የያዘ መጠጥ የሚጠጡት?	በየቀኑ ማለት ይቻላል.....................1<br>ቢያንስ በሳምንት አንድ ጊዜ............2<br>በሳምንት ከአንድ ጊዜ በታች...........3<br>ቢያንስ በወር አንድ ጊዜ.................4	
503	ባለቤትዎ/ የፍቅር ጓደኛዎ አሁን ከእርስዎ ጋር ነው ወይንስ ሌላ ቦታ ነው የሚኖረው?	ከእኔ ጋር ነው የሚኖረዉ................1<br>ሌላ ቦታ ነው የሚኖረው................2	

	(ላገባ ብቻ)		
504	ባለቤትዎ/ የፍቅር ጓደኛዎ ለስራ በተደጋጋሚ ወደ ሌላ ቦታ ይጓዛል?	አዎ...1<br>አይጓዝም 2	
505	በአለፉት ሶስት ወራት ውስጥ አዲስ የትዳር አጋር / የፍቅር ጓደኛ አለዎት?	አዎ...1<br>የለኝም..2	
506	ከባለቤትዎ/ ከፍቅር ጓደኛዎ ጋር ምን ያህል ጊዜ አብረዉ ኖረዋል?	ጊዜ_______	
507	ባለቤትዎ/የፍቅር ጓደኛዎ አልኮል የያዘ መጠጥ ጠጥቶ ያዉቃል? (ለምሳሌ ጠላ ፣ አረቂ፣ ጠጅ፣ ቢራ ፣ ወይን ወዘተ)	አወ...1<br>አያዉቅም.................................2<br>አላዉቅም...............................88 →	509
508	ባለቤትዎ/የፍቅር ጓደኛዎ በ ምን ያህል ጊዜ ውስጥ ነው አልኮል የያዘ መጠጥ የሚጠጣው?	በየቀኑ ማለት ይቻላል......................1<br>ቢያንስ በሳምንት አንድ ጊዜ.............2<br>በሳምንት ከአንድ ጊዜ በታች............3<br>ቢያንስ በወር አንድ ጊዜ..................4	
509	የባለቤትዎን/ የፍቅር ጓደኛዎን የኤች አይ ቪ ሁኔታ የቅርብ ዕውቀት አለዎት?	አዎ..1<br>የለኝም...2 →	511
510	የባለቤትዎ/ የፍቅር ጓደኛዎ የኤች አይ ቪ ሁኔታ ምንድን ነው?	ኤች አይ ቪ አለበት ....1<br>ኤች አይ ቪ የለበትም 2	
511	አሁን አንዳንድ አስፈላጊ የሆኑ የሂዎት ጉዳዮችን በተሻለ ለመረዳት ወሲባዊ ሂወትዎን የሚዳስሱ የተወሰኑ ጥያቄዎችን መጠየቅ እፈልጋለሁ፡ መልሰችዎ ሙሉ በሙሉ ሚስጢራዊ አና ለማንም የማይነገሩ መሆናቸዉን በድጋሜ ላረጋግጥለዎት እወዳለሁ፡ መልስ መስጠት የማይፈልጉትን ማንኛዉንም ጥያቄ አሳዉቁን እና ወደ ሚቀጥለዉጥያቄ እንሄዳለን። የመጀመሪያውን ግብረ ስጋ ግንኙነት በፈፀሙበት ወቅት እድሜዎ ስንት ነበር?	አመት_______	
512	ለመጨረሻ ጊዜ የግብረ-ስጋ ግንኙነት የፈፀሙት መቸ ነበር?	ከ________ቀናት በፊት<br>ከ________ሳምንታት በፊት<br>ከ________ወራት በፊት	
513	ለመጨረሻ ጊዜ የግብረ-ስጋ ግንኙነት በፈፀሙበት ወቅት ኮንዶም ተጠቅመዋል?	አወ...1<br>አልተጠቀምኩም.........................2<br>እርግጠኛኛ አይደለሁም...............3	
514	ከባለቤትዎ/ከፍቅር ጓደኛዎ በተጨማሪ ከሌላ ሰዉ ጋር የግብረ-ስጋ ግንኙነት ፈፅመዋል?	አወ...1<br>አልፈፀምኩም............................2 →	516

515	በጠቅላላዉ ምን ያህል ሰዎች ጋር የግብረ-ስጋ ግንኙነት ፈፅመዋል?	ሰዎች ጋር________<br>አላውቅም.................................88	
516	ባለቤትዎ/ የፍቅር ጓደኛዎ ልላ የትዳር አጋር አለው? (ወይንም እንዳገባ ሆኖ ከሌላ ሴት ጋር ይኖራል)?	አዎ..1<br>የለውም....................................2<br>አላውቅም 88	
517	ባለቤትዎ / የፍቅር ጓደኛዎ ሌላ ወሲባዊ አጋር አለው ብለው ይጠረጥራሉ?	1.አዎ.......................................1<br>2.አልጠረጥርም.........................2<br>3.አላውቅም............................88	
518	ባለቤትዎ ወይም የፍቅር ጓደኛዎ ስሜትዎን የሚጎዱ ነገሮችን ያደርጋል? (ለምሳሌ ስድብ፤ ማጣጣል፤ ለመጉዳት ማስፈራራት፤ መጮህ፤ መርገም፤ ለራስሽ መጥፎ ስሜት እንዲሰማሽ ማድረግ)	በፍጹም...1<br>አንዳንድ ጊዜ.....................................2<br>ብዙዉን ጊዜ......................................3<br>ሁልጊዜ ለማለት ይቻላል....................4	
519	ባለቤትዎ ወይም የፍቅር ጓደኛዎ አካላዊ ጥቃት አድርሶቦት ያውቃል?(ለምሳሌ ፤በጥፊ መምታት፣በካልቾ መምታት፣መገፍተር)	በፍጹም...1<br>አንዳንድ ጊዜ......................................2<br>ብዙዉን ጊዜ......................................3<br>ሁልጊዜ ለማለት ይቻላል.....................4	
520	ባለቤትዎ ወይም የፍቅር ጓደኛዎ ጾታዊ ጥቃት ፈጽሞቦት ያውቃል? (ለምሳሌ ባልፈለግሺበት ጊዜ የግብረ-ሥጋ ግንኙነት እንዲፈጽሙ ማስገደድ)?	በፍጹም...1<br>አንዳንድ ጊዜ......................................2<br>ብዙዉን ጊዜ......................................3<br>ሁልጊዜ ለማለት ይቻላል.....................4	

ክፍል ሰባት ፡- የሴቷ የኤች አይ ቪ ሁኔታ

701	ድጋሜ ምርመራ ከተደረገላት በኋላ ያለው የኤች አይ ቪ ውጤት	ኤች አይ ቪ ያልተገኘባት................1<br>ኤች አይ ቪ የተገኘባት.....................2	

ስለሰጡን ጊዜ እናመሰግናለን።

የመረጃሰብሳቢውስም______________________________ፊርማ____________________

የተቆጣጣሪውስም______________________________ ፊርማ____________________
